# Reciprocal space slicing: A time-efficient approach to femtosecond x-ray diffraction

**DOI:** 10.1063/4.0000040

**Published:** 2021-01-21

**Authors:** S. P. Zeuschner, M. Mattern, J.-E. Pudell, A. von Reppert, M. Rössle, W. Leitenberger, J. Schwarzkopf, J. E. Boschker, M. Herzog, M. Bargheer

**Affiliations:** 1 Institut für Physik und Astronomie, Universität Potsdam, 14476 Potsdam, Germany; 2 Helmholtz-Zentrum Berlin für Materialien und Energie GmbH, Wilhelm-Conrad-Röntgen Campus, BESSY II, 12489 Berlin, Germany; 3 Leibniz-Institut für Kristallzüchtung, 12489 Berlin, Germany

## Abstract

An experimental technique that allows faster assessment of out-of-plane strain dynamics of thin film heterostructures via x-ray diffraction is presented. In contrast to conventional high-speed reciprocal space-mapping setups, our approach reduces the measurement time drastically due to a fixed measurement geometry with a position-sensitive detector. This means that neither the incident (*ω*) nor the exit (2θ) diffraction angle is scanned during the strain assessment via x-ray diffraction. Shifts of diffraction peaks on the fixed x-ray area detector originate from an out-of-plane strain within the sample. Quantitative strain assessment requires the determination of a factor relating the observed shift to the change in the reciprocal lattice vector. The factor depends only on the widths of the peak along certain directions in reciprocal space, the diffraction angle of the studied reflection, and the resolution of the instrumental setup. We provide a full theoretical explanation and exemplify the concept with picosecond strain dynamics of a thin layer of NbO_2_.

## I. INTRODUCTION

Modern crystallography and strain assessment at the nanoscale cannot be imagined without x-ray diffraction. This nondestructive and widely available tool to determine interatomic distances in crystalline specimen has been proven to be particularly useful in the ultrafast dynamics of condensed matter.^1–4^ Technological progress relies on the development of novel and faster procedures to transfer energy between subsystems and a decreasing size of the devices. This implies the importance of quantifying strain in nanoscale specimens of technologically relevant materials on the picosecond timescale.^5–10^

Ultrafast x-ray diffraction (UXRD) setups are sensitive to changes in the diffraction pattern, which map out the reciprocal space (RS) of the specimen in which lattice dynamics have been triggered.^11^ To access details of the crystalline order in the reciprocal space, all the diffracted intensity needs to be spatially quantified as a function of the angular relation between the incoming x-ray beam, the sample, and the detector.^11,12^ Scanning both angles of a point detector and the sample takes a large amount of time, especially if multiple reciprocal space maps (RSMs) need to be recorded for time-resolved measurements.

With the introduction of position-sensitive detectors, i.e., pixel-area or pixel-line detectors, it became possible to measure the diffracted intensity on a linear subset of the reciprocal space simultaneously.^12–14^ Consequently, the time for full reciprocal space mapping decreased drastically, e.g., for time-resolved strain assessment. A detection scheme with a fixed detector has been used in the context of high repetition rate UXRD experiments at synchrotrons.^15^

In this paper, we discuss an experimental method to determine the strain perpendicular to the surface of nanoscale heterostructures, which reduces the acquisition time even more. The data acquisition routine, which we call reciprocal space slicing (RSS), constantly monitors just a subset of the reciprocal space of a specimen with an area detector and a fixed diffraction geometry (see Sec. II). We analyze theoretically that reciprocal space slicing is sufficient to monitor strain dynamics perpendicular to the surface of most thin, layered specimen without scanning the diffraction angles. This applies for diffraction setups with monochromatic and parallel or convergent x-rays (see Sec. III A or Sec. IV A, respectively).

We test our theory experimentally at the KMC-3 XPP Beamline at the BESSY II synchrotron of the HZB^16,17^ for parallel x-rays in Sec. III B. For a convergent x-ray beam, reciprocal space slicing is validated experimentally at the femtosecond x-ray diffraction setup with a laser-based plasma x-ray source (PXS)^18,19^ (see Sec. IV B). With both setups, we demonstrate the slicing by examination of a sample fabricated by pulsed laser deposition a thin layer of NbO_2_ on top of a TiO_2_ substrate.^20^ NbO_2_ exhibits an insulator-metal phase transition, accompanied by a transition in the crystalline ordering at 1070 K.^21^ This renders NbO_2_ and its alloys a promising material class for electrical switching and even memory applications at high temperatures.^22–24^ Future ultrafast strain assessment during the phase transition using UXRD may reveal novel insight into the transition and promote application development in electronics, complementing all-optical studies.^25^ For this publication, however, the NbO_2_ sample was mainly selected because of its particular shape in reciprocal space, displaying a high-contrast twofold nature of the Bragg reflections, due to a particular domain structure resulting in a large discrepancy of the structural, in-plane coherence length. This sample is, thus, ideal to demonstrate the advantages and limitations of reciprocal space slicing for narrow and broad Bragg reflections in a single measurement.

## II. RECIPROCAL SPACE SLICING

Generally, the measured intensity in reciprocal space ($I(\vec{Q})$) is a convolution of the reciprocal space (*RS*) of the specimen and the instrument's resolution area (*RA*),^11^

$$I(\vec{Q})=(RS*RA)(\vec{Q}).$$ (1)

The shape of the resolution area is determined by the energy distribution and the trajectories of the x-ray photons used for diffraction. In reciprocal space, this translates into the length and directions of the incident x-ray wave vectors ${\vec{k}}_{\text{in}}$. Since *RA* is different for the two presented experimental setups in this paper, we evaluate the role of *RA* in Secs. III A and IV A. But the shape of the reciprocal space is determined by the coherence length of the scattering periodic structure of the specimen.^11^ As we use the same sample throughout this paper, *RS* is the same and is modeled as follows.

**FIG. 1.**
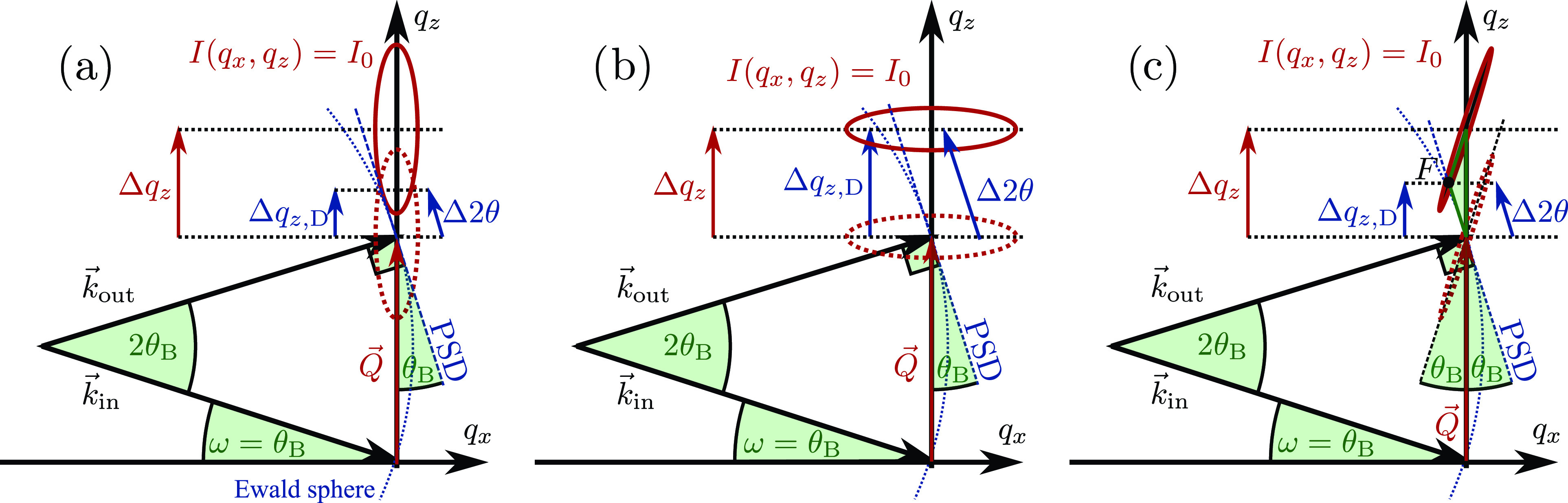
Schematic sketch of a symmetric diffraction geometry set to the Bragg angle $\theta_{B}$ for different shaped intensity distributions surrounding $\vec{Q}=\vec{G}$. *q_x_* is aligned along the sample surface. The red dashed and solid ellipses represent contour lines of the intensity distribution $I(q_{x},q_{z})$ surrounding $\vec{G}$ before and after the shift along *q_z_*, respectively. The dotted blue line represents the Ewald sphere on which the detector is positioned. We approximate the arc by a straight line, indicated by the dashed blue line, labeled PSD. (a) *I* is elongated along *q_z_*, which results in a small visible Δ2θ on the detector. (b) *I* is elongated along *q_x_*, which leads to $\Delta q_{z,D}\approx \Delta q_{z}$. (c) $2\Delta q_{z,D}\approx \Delta q_{z}$, for reflections with high crystalline quality at the PXS setup (see Sec. IV).

For thin films, the reciprocal space $RS(\vec{Q})$ in the vicinity of $\vec{G}$ can be approximated by a Gaussian function. In this paper, we visualize the 3D reciprocal space by a 2D projection onto the $q_{x}/q_{z}$-plane since we only discriminate between the in- and out-of-plane directions. Here, *q_z_* is aligned perpendicular and *q_x_* parallel to the sample surface and diffraction plane. Thus, we model $RS(\vec{Q})$ of a thin layer, by a 2D-Gaussian function,

$$RS(q_{x},q_{z})=A_{\text{RS}}\;\text{exp}\;\left(-\frac{{\left(q_{x}-g_{x}\right)}^{2}}{2\sigma_{x}^{2}}-\frac{{\left(q_{z}-g_{z}-\Delta q_{z}\right)}^{2}}{2\sigma_{z}^{2}}\right),$$ (2)

where *σ_x_* and *σ_z_* are the widths along *q_x_* and *q_z_*, which are inversely proportional to the in-plane and out-of-plane coherence lengths, respectively. The amplitude $A_{\text{RS}}$ is proportional to the structure factor, and *g_x_* and *g_z_* are the components of $\vec{G}$, which has an absolute value that is inversely proportional to the lattice spacing in real space. In our case, *g_x_* is close to 0, as we analyze lattice planes parallel to the sample surface. The shift of $\vec{G}$ resulting from out-of-plane strain is given by $\Delta q_{z}$. A contour line of this particular intensity in the $q_{x}/q_{z}$-plane is an ellipse, in which both semi-axis lengths correspond to the widths *σ_x_* and *σ_z_*. In Figs. 1(a) and 1(b), $I(q_{x},q_{z})$ is visualized for opposing $\sigma_{x}/\sigma_{z}$ ratios by ellipses to provide an intuitive geometric approach.

In a symmetric diffraction geometry, with the center of the position sensitive detector set to twice the Bragg angle of the probed material ($2\theta_{B}$), the detector intersects the corresponding reciprocal lattice point $\vec{G}$, which is positioned at $\vec{G}=\vec{Q}:={\vec{k}}_{\text{out}}-{\vec{k}}_{\text{in}}$ in the reciprocal space map (RSM).^11,19^ This means that the area detector slices the intensity distribution in reciprocal space (*RS*) in the center (Fig. 1), as the pixels record a large 2θ range of the diffracted signal.

Strain of the specimen perpendicular to the surface results in a change in the *q_z_*-component of $\vec{G}$. Conventionally, this is detected by comparing an RSM of the strained sample with an RSM of a reference state. In this process, the RSMs are assembled by the reciprocal space slices recorded at different combinations of the diffraction angle *ω* and the 2θ range of the detector area.^11^ Here, *ω* is the angle between ${\vec{k}}_{\text{in}}$ and the lattice planes of the corresponding $\vec{G}$, which are chosen to be parallel to the sample surface in this paper. 2θ denotes the angle enclosed by ${\vec{k}}_{\text{in}}$ and ${\vec{k}}_{\text{out}}$.

In contrast to full reciprocal space mapping, the diffraction geometry is fixed during reciprocal space slicing, which means, in particular, that *ω* and 2θ are set and fixed to $\theta_{B}$ and $2\theta_{B}$. This decreases the measurement time significantly, because no angular scans are required. The resulting $\Delta q_{z}$ of a strained material manifests itself in a shift of the diffracted intensity distribution on the position sensitive detector (Δ2θ) (see Fig. 1). The projection of Δ2θ onto the *q_z_* axis $\Delta q_{z,D}$ is proportional to $\Delta q_{z}$, and the proportionality factor *S* depends on the diffraction geometry, the resolution area of the experimental setup (*RA*), and the reciprocal space of the specimen (*RS*) in proximity to $\vec{G}$. Consequently, we are able to determine the strain *η* by just scaling the shift of the 1D intensity distribution on the detector with the factor *S*. Since the detector area is tangent to the 2θ-circle, the strain *η* is proportional to the change in the diffraction angle Δ2θ on the detector and *S*,

$$\eta =-\frac{\Delta q_{z}}{|\vec{G}|+\Delta q_{z}}=-S\frac{\Delta q_{z,D}}{|\vec{G}|+\Delta q_{z}}=-S\frac{\Delta 2\theta }{2}\text{cot}(\theta_{B}^{\prime }),$$ (3)

where $\theta_{B}^{\prime }$ is the Bragg angle after the shift occurred. For small strains below a few %, we can approximate $\theta_{B}^{\prime }\approx \theta_{B}$ and $\left|\vec{G}|+\Delta q_{z}\approx |\vec{G}\right|$.

In Secs. III–IV, we will give a quantitative evaluation of the functional dependence of the desired observable $\Delta q_{z}$ on the shift observed on the detector Δ2θ. For this, we put in different *RA*s for the two different experimental setups and model the intensity distribution on the detector as a function of the shift $\Delta q_{z}$.

## III. RECIPROCAL SPACE SLICING AT SYNCHROTRONS

### A. The role of position sensitive detectors in RSS

First, we assume a resolution area, which corresponds to a monochromatic and parallel x-ray beam, which means that the resolution area can be approximated by a *δ*-function in the wave vector and energy space. Thus, the RSM is equal to the reciprocal space of the specimen: $I(\vec{Q})=RS(\vec{Q})$, according to Eq. (2).

The intensity distribution measured by a detector line ($I_{D}$) is a one-dimensional subset of the intensity distribution $I(q_{x},q_{z})$, namely, an arc with the radius $k_{\text{in}}$, i.e., a fraction of the Ewald sphere for symmetric diffraction.^11,19^ We approximate this arc as a line, as the size of the intensity distribution around $\vec{G}$ is typically comparably small with respect to the wave vector. Even for a minimal coherence length of $L_{c}=\pi /(\sqrt{2\;\text{ln}\;2}\sigma_{q})=1$ nm, the width $\sigma_{q}\approx 0.3$ Å^–1^ of the distribution $RS(\vec{Q})$ in the corresponding direction is an order of magnitude smaller than usual hard x-ray wave vectors that are on the order of $|{\vec{k}}_{\text{in}}|\approx 4$ Å^–1^ (8 keV). With this, the detector can be described by a linear parametric function, which defines a subset of the reciprocal space via Eq. (4), which is indicated by a dashed blue line in Fig. 1,

$$q_{z}=-\frac{q_{x}}{\;\text{tan}\;(\theta_{B})}+g_{z}.$$ (4)

Since this defines all pairs (*q_x_*, *q_z_*) at which the detector measures the intensity, we substitute *q_x_* in Eq. (2) with Eq. (4) to get the measured intensity on the detector line $I_{D}$ as a function of *q_z_* only, which is again a 1D Gaussian function,

$$\begin{array}{c} I_{D}(q_{z}):=I(-(q_{z}-g_{z})\;\text{tan}\;(\theta_{B}),q_{z})\\ =A_{RS}\;\text{exp}\;\left(-\frac{\;\text{tan}\;{\left(\theta_{B}\right)}^{2}{\left(q_{z}-g_{z}\right)}^{2}}{2\sigma_{x}^{2}}-\frac{{\left(q_{z}-g_{z}-\Delta q_{z}\right)}^{2}}{2\sigma_{z}^{2}}\right)\\ \overset{!}{=}A_{D}\;\text{exp}\;\left(-\frac{{\left(q_{z}-g_{z}-\Delta q_{z,D}\right)}^{2}}{2\sigma_{D}^{2}}\right), \end{array}$$ (5)

where $A_{D}$ is a scaled amplitude, $\sigma_{D}$ is the width, and $\Delta q_{z,D}$ is the shift of the intensity distribution on the detector line projected onto the *q_z_* axis. The relation between the strain-induced change of $\vec{G}$ ($\Delta q_{z}$) and the shift that is measured ($\Delta q_{z,D}$) is, therefore, given by

$$\Delta q_{z}=\Delta q_{z,D}\underset{=:S}{\underset{︸}{\left(1+\frac{\sigma_{z}^{2}}{\sigma_{x}^{2}}\text{tan}\;{\left(\theta_{B}\right)}^{2}\right)}}.$$ (6)

In the experiments with a fixed diffraction geometry and a *δ*-shaped instrument function, it is thus possible to derive the change in $\vec{G}$ and hence, the strain by just scaling the shift of the 1D intensity distribution on the detector with the factor *S*. However, this is only applicable for broad intensity distributions in reciprocal space. For very narrow Bragg reflections, e.g., as typical of substrates, even small shifts along *q_z_* lead to a massive intensity loss on the fixed detector so that the diffracted intensity quickly becomes impossible to detect.

In Figs. 1(a) and 1(b), we display the two limits of this result with which we illustrate several important implications. In (a), $\sigma_{x}<\sigma_{z}$, which is the case for a single-crystalline thin film, results in a rather large intensity loss but only a small shift of the intensity distribution on the detector $\Delta q_{z,D}$ compared to the real shift of the intensity maximum $\Delta q_{z}$. In (b), on the other hand, where $\sigma_{x}>\sigma_{z}$, which is the case for columnar growing films, the observed shift on the detector $\Delta q_{z,D}$ is basically equal to $\Delta q_{z}$ and the amplitude does not change significantly either. Figure 1 also illustrates that the discrepancies between $\Delta q_{z,D}$ and $\Delta q_{z}$ become more pronounced with increasing diffraction angles, e.g., at higher diffraction orders.

### B. Thermally induced strain measured with RSS

In this section, we evaluate the negative thermal expansion of the 75 nm thin NbO_2_ layer on top of a TiO_2_ substrate at the KMC-3 XPP beamline at the BESSY II synchrotron of the HZB,^16,17^ using the reciprocal space slicing theory described in Sec. II.

**FIG. 2.**
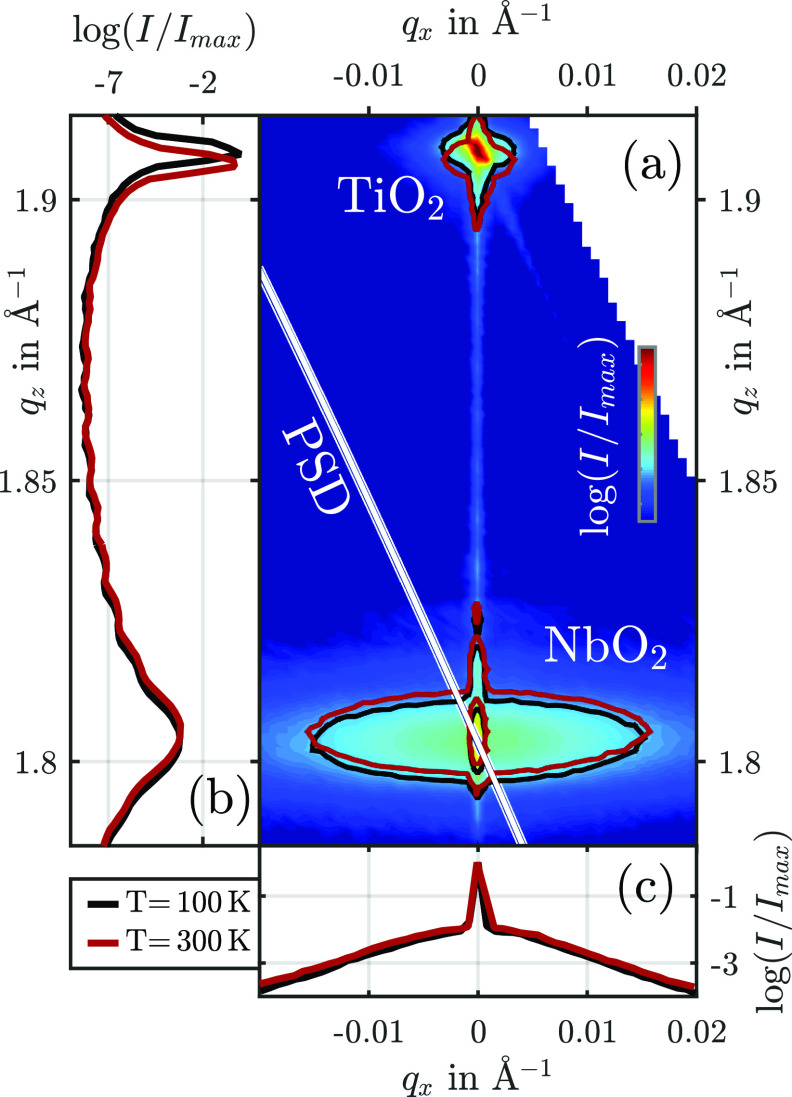
(a) RSM of the sample recorded at 100 K at the KMC-3 XPP beamline in the vicinity of the (200) NbO_2_-layer and (220) TiO_2_-substrate Bragg reflections. The black and red lines are contour lines of the RSM at 100 K and at 300 K, respectively. (b) and (c) Projections of the RSM onto the *q_z_*- and *q_x_*-axis, respectively. The *q_x_*-projection contains only the RSM in close proximity to the NbO_2_ reflection (1.78 ${Å}^{-1}<q_{z}<$ 1.83 Å^−1^). The white line indicates the linear subset of the reciprocal space, which is measured simultaneously by the detector (PSD) and defined by Eq. (4). The intensity distribution on the detector is shown in Fig. 3.

The thin layer of NbO_2_ exhibits a tetragonal crystal structure where the (100) direction, which coincides with the (110) direction of the rutile ordered TiO_2_, is aligned out-of-plane, i.e., parallel to *q_z_*. We scanned the full reciprocal space in proximity to the (200) and (220) Bragg reflections of NbO_2_ and TiO_2_, respectively, with 8 keV parallel x-rays and an area detector (*Pilatus 100 K* from Dectris). A projection onto the $q_{x}/q_{z}$-plane at a sample temperature of 100 K is displayed in Fig. 2(a). A projection of the intensity of both reflections onto the *q_z_*-axis is displayed in (b), and the projection of the NbO_2_ reflection onto the *q_x_*-axis is displayed in (c). The black contour lines and graphs correspond to the RSM recorded at 100 K and the red lines to the RSM recorded at 300 K.

The Bragg reflections yield the following information about the crystalline structure of the sample. The reflection of the TiO_2_ substrate is narrow in reciprocal space, only deformed and broadened by the crystal truncation rod, analyzer and monochromator streaks, and thermal diffuse scattering.^11^ The NbO_2_ reflection, on the other hand, reveals two contributions, indicated by the broad (b) horizontally elongated and narrow (n) vertically aligned ellipsoidal contour lines for two different intensities of the RSM, shown in Fig. 2(b). The projections onto the *q_x_* and *q_z_* axes allow the determination of the widths along the two directions by Gaussian fits and, consequently, the quantification of the coherence lengths parallel and perpendicular to the surface. We find a single width of $\sigma_{z}=0.004$ Å^–1^ in the *q_z_* direction and the widths of $\sigma_{x,b}=0.009$ Å^–1^ for the broad part and an upper limit of $\sigma_{x,n}=0.0003$ Å^–1^ for the narrow portion along *q_x_*. This corresponds to an out-of-plane coherence length of $\xi_{z}=66$ nm, which is close to the layer thickness of 75 nm. Parallel to the surface, the widths correspond to coherence lengths of $\xi_{b}=30$ nm and at least $\xi_{n}=900$ nm. The latter can be even larger, but we are limited by the scanning resolution. The occurrence of two coherence lengths parallel to the surface is associated with lattice mismatches of TiO_2_ and NbO_2_. The *c*-axes of both tetragonal materials are aligned with a small lattice mismatch of just 1%. This leads to a well-ordered crystalline structure of NbO_2_ and a large coherence length parallel to this direction. Along the in-plane direction perpendicular to the *c*-axis, the lattice mismatch is considerably larger with over 5%. This leads to strong distortions in NbO_2_ and a small coherence length. AFM measurements of the surface support this interpretation by revealing domains at the nanometer length scale.^20^ Since the x-ray probe spot on the specimen measures tens to hundreds of micrometers in both directions, the signal of the diffracted intensity is a lateral average of the domains and thus, contains both contributions.

The change of the contour lines and graphs from black to red in Fig. 2 exemplify the lattice response of the TiO_2_/NbO_2_ heterostructure to static heating. A rise in temperature from 100 K to 300 K leads to the expected thermal expansion of TiO_2_, which we measure directly by the shift of the (220) reflection of TiO_2_ to lower *q_z_*.^26^ Simultaneously, both contributions of the NbO_2_ (200) reflection shift to larger *q_z_* with increasing temperature, which corresponds to the reported negative thermal expansion of NbO_2_ along the *a*-axis of the tetragonal unit cell below room temperature.^27^ The *q_z_* shift due to the contraction can be quantified by the comparison of two RSMs and the projection onto the *q_z_* axis [see Fig. 2(b)].

**FIG. 3.**
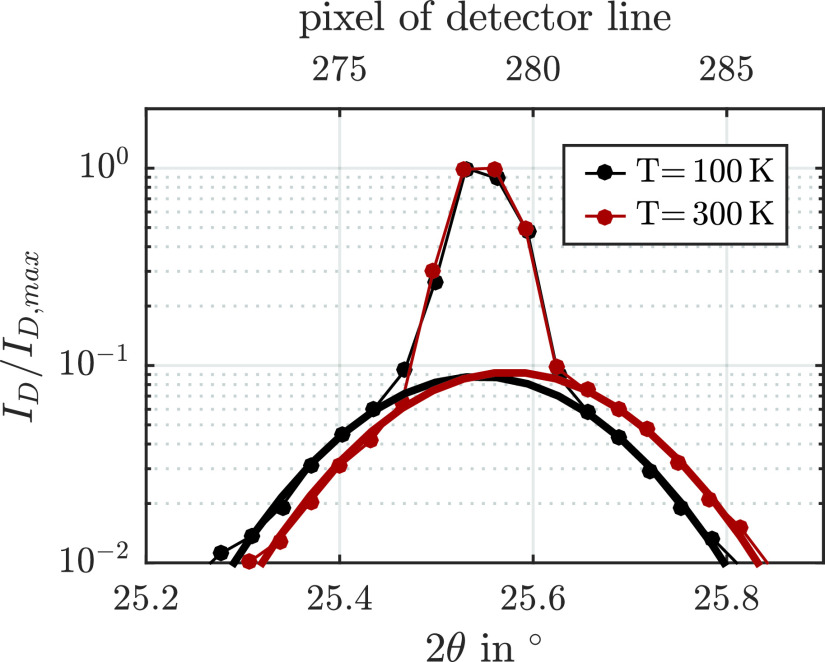
Relative intensity distribution on the area detector after integration perpendicular to the diffraction plane for 100 and 300 K. The position of the detector pixels is transformed to 2θ, according to the diffraction geometry. The data are fitted by a broad Gaussian function to indicate the larger shift of the broad component of the NbO_2_ (200) reflection in comparison to the narrow contribution that remains almost unchanged.

We now discuss reciprocal space slicing as a faster alternative for $\Delta q_{z}$ assessments, which uses just one detector image of the RSM scan at 100 and 300 K. The two detector images for the two temperatures are displayed in Fig. 3 after integration of the intensity on the detector area perpendicular to the diffraction plane. The diffraction patterns on the detector exhibit the two contributions of the NbO_2_ reflection, but only the broad part reveals a visible shift along the diffraction angle 2θ with increasing temperature. This finding agrees with the modeling in Sec. III A since the visible shift of the diffraction pattern on the detector line Δ2θ is proportional to the strain, but the proportionality factor *S* depends on the shape of the intensity distribution via Eq. (6). With the widths of the two contributions, we get $S_{b}=1.01$ for the broad and $S_{n}=11$ for the narrow component. Since the real thermally induced strain is expected to be identical for both contributions, Δ2θ must be more than ten times smaller for the narrow component of the NbO_2_ reflection than for the broad component [see Eq. (3)]. This is consistent with the data in Fig. 3. Additionally, we crosschecked the quantitative agreement of the strain determined by conventional full reciprocal space mapping and slicing. $\Delta q_{z}$, determined with RSM data in Fig. 2, is, in fact, equal to $S_{b}\Delta q_{z,D}$.

This exemplifies that reciprocal space slicing can be a very effective way to determine out-of-plane strain if the intensity distribution in the vicinity of $\vec{G}$ is comparably broad along *q_x_* compared to *q_z_*, which is the limiting case depicted in Fig. 1(b). It also illustrates that shifts of $\vec{G}$ with a surrounding intensity distribution, which are very narrow along *q_x_* compared to *q_z_*, can be much better quantified with full reciprocal space mapping. Thus, slicing with highly parallel beams at synchrotrons is only effective for thin layers with in-plane coherence lengths comparable to the layer thickness, i.e., specimen with noticeable mosaicity or a domain structure.

## IV. RECIPROCAL SPACE SLICING WITH A COMPLEX RESOLUTION AREA

### A. The role of convergent and polychromatic x-rays in RSS

The resolution area can have a rather complex shape in many experimental setups. In this section, we illustrate the application of reciprocal space slicing for the plasma x-ray source (PXS) at the University of Potsdam, which is optimized for ultrafast x-ray diffraction (UXRD) with a pulse length on the order of 200 fs.^18^ The probing x-ray pulses are composed of the K_*α*1_ and K_*α*2_ lines of copper and are focused onto the specimen by a Montel optic (Incoatec ELM45).^28^ The diffracted signal is detected by an area detector (*Pilatus 100 K* from Dectris).^19^ For static measurements, the PXS can be replaced by a microfocus x-ray tube (*UltraBright 96000* from Oxford instruments) with the focus positioned where the femtosecond x-rays emerge.

In both cases, the resolution area of this setup is described by the sum of two 2D Gaussian functions.^19^ The Gaussian doublet is separated by 0.25% of the chosen scattering vector $\vec{Q}$ with respect to the *q_z_* axis due to the energy difference of the K_*α*1_ and K_*α*2_ lines and is broadened along *q_z_* by the natural linewidth of these characteristic lines. The major axis of the Gaussian doublet is determined by the convergence of 0.3° from the x-ray optic. The pixel size of the detector limits the resolution of the detected x-rays along the minor axis of the Gaussian doublet. This is associated with an uncertainty of the diffraction angle, which can reach from 0.1° to 0.005°, depending on the sample-detector distance of 0.1 to 2 m. In reciprocal space, the doublet is rotated by the diffraction angle of the incident x-rays *ω* [see Fig. 1(c)].^19^

As is shown in the Appendix, the intensity distribution $I_{D}$ of an elongated $\vec{G}$ measured along the slice of the detector is again a Gaussian function and can be written as

$$I_{D}=A_{I_{D}}\;\text{exp}\;\left(-\frac{{\left(q_{z}-\frac{\Delta q_{z}}{S}\right)}^{2}}{2\sigma_{I_{D}}^{2}}\right),$$ (7)

where $A_{I_{D}}$ is the amplitude and $\sigma_{I_{D}}$ is the width of the intensity distribution of the detector projected onto the *q_z_* axis. The scaling factor *S* connects the measured *q_z_* shift on the detector ($\Delta q_{z,D}$) with the real shift $\Delta q_{z}$ of $\vec{G}$, similar to Eq. (6). In this case, *S* depends on the parameters of the resolution area as well. For symmetric diffraction geometries with $\omega =\theta_{B}$, *S* is given by Eq. (8) that contains two simple implications if we evaluate two opposite limiting cases.

On the one hand, we can assume very broad intensity distributions in the reciprocal space of the specimen in proximity to $\vec{G}$, i.e., $\sigma_{x},\sigma_{z}\gg \sigma_{\text{RA},x},\sigma_{\text{RA},z}$. This is the case for samples that exhibit small coherence lengths in- and out-of-plane, in particular, thin films with noticeable mosaicity. Then, Eq. (8) reduces to the definition of *S* in Eq. (6), which is expected, as the previously assumed *δ*-like resolution area is always narrower than any other feature in the reciprocal space of the specimen.

$$S:=\frac{\sigma_{\text{RA},x}^{2}-\sigma_{z}^{2}+\frac{\sigma_{z}^{2}}{\;\text{cos}\;{\left(\theta_{B}\right)}^{2}}+\frac{\sigma_{\text{RA},x}^{2}}{\;\text{cos}\;{\left(\theta_{B}\right)}^{2}}-4\;\text{sin}\;(\theta_{B})(\sigma_{\text{RA},x}^{2}-\sigma_{\text{RA},z}^{2})}{\sigma_{x}^{2}+\sigma_{x}^{2}-2\;\text{sin}\;(\theta_{B})(\sigma_{\text{RA},x}^{2}-\sigma_{\text{RA},z}^{2})}.$$ (8)

On the other hand, we can assume a reciprocal space, which exhibits intensity distributions surrounding $\vec{G}$ that are far narrower than the resolution area of the PXS setup. This is the case for samples with large coherence lengths, i.e., substrates or films with high-quality crystallinity. In that case, the expression in Eq. (8) becomes

$$S=\frac{2\;\text{sin}\;{\left(\theta_{B}\right)}^{2}(\sigma_{RA,x}^{2}-\sigma_{RA,z}^{2})-\frac{1}{2}\sigma_{RA,x}^{2}/\;\text{cos}\;{\left(\theta_{B}\right)}^{2}}{\;\text{sin}\;{\left(\theta_{B}\right)}^{2}(\sigma_{RA,x}^{2}-\sigma_{RA,z}^{2})-\sigma_{RA,x}^{2}/2},$$ (9)

which converges to two for $\sigma_{\text{RA},z}\gg \sigma_{\text{RA},x}$, as it is the case for the PXS setup that has $\sigma_{\text{RA},z}\approx 20\times \sigma_{\text{RA},x}$.^19^ This limit of a factor of 2 can also be motivated via a geometrical reasoning shown in Fig. 1(c). A contour line of the measured intensity distribution from a substrate is sketched in red, neglecting the twofold nature of the resolution area at the PXS. The semi-major axis of this ellipse is related to $\sigma_{\text{RA},z}$ and the semi-minor axis to $\sigma_{\text{RA},x}$. The ellipse is tilted clockwise by the angle $\theta_{B}$ with respect to the *q_z_* axis, whereas the detector line is inclined by the same angle, but counterclockwise. In this symmetric diffraction geometry, the semi-major axis of the ellipse, the detector line, and the *q_z_* axis constitute an isosceles triangle (green). Therefore, the *q_z_* projection of the intersection point *F* of the semi-major axis and the detector line always is exactly half of the true shift of $\vec{G}$.

### B. Picosecond strain dynamics with RSS

In this section, we present a complete evaluation of picosecond strain dynamics of the sample in the context of reciprocal space slicing. To employ the slicing technique, we first record a full reciprocal space map (RSM) of the specimen without optical excitation to determine the shape of the resolution area and the reciprocal space in proximity to $\vec{G}$ of the thin NbO_2_ layer. Ideally, the latter is determined at a synchrotron-based diffraction setup with very high angular resolution (see Fig. 2 for the RSM with a very small resolution area). Clearly, the TiO_2_ substrate reflection is much sharper than the thin layer NbO_2_ reflection, which is composed of two contributions with very different widths along *q_x_*. Ultrafast diffraction experiments combining a time resolution of 100 fs with such a small resolution area can only be recorded at free-electron lasers or femto-slicing beamlines. At synchrotrons, the time resolution is typically limited to 100 ps. Here, we discuss a table top femtosecond x-ray diffraction setup driven by a PXS.

The transient response of the sample is probed with a 200 fs x-ray pulse composed of the Cu K_*α*_ doublet in the convergent beam geometry described in Sec. IV A. For ultrafast x-ray diffraction experiments, we excite the sample with 100 fs pulses centered around 800 nm, at an incident pump-energy density of 10 mJ/cm^2^. The time resolution of this setup approaches the state-of-the-art at free electron lasers, however, with many orders of magnitude less brilliance and with a much larger resolution area. We shall see in the following that the broad resolution area may be advantageous for the presented technique.

**FIG. 4.**
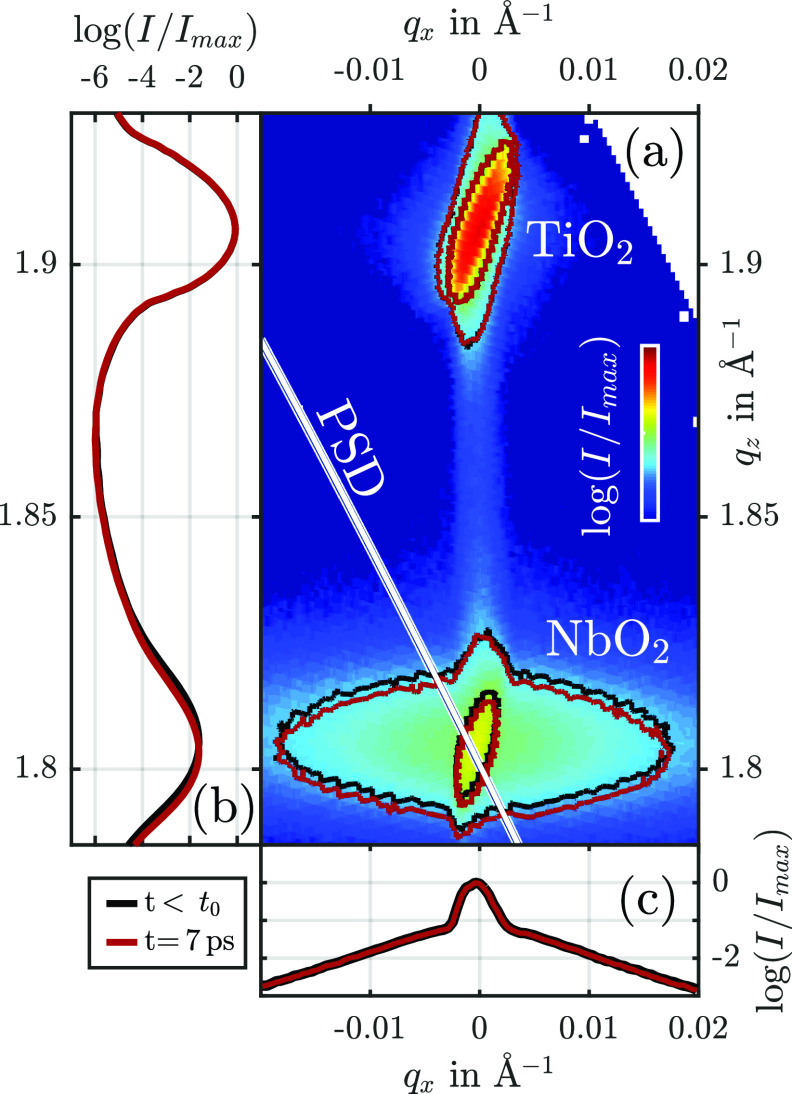
RSM (a) of the sample with projections onto *q_z_*- (b) and *q_x_*-axes (c). The TiO_2_ (110) substrate peak is visible at *q_z_* = 1.91 Å^–1^ and the NbO_2_ (100) layer peak at *q_z_* = 1.82 Å^–1^. The ellipses in (a) are contour lines of the RSM before (black) and 7 ps after excitation (red). Accordingly, the projections onto the axes in (b) and (c) are taken from the RSM before and after excitation. The white line indicates the linear subset of the reciprocal space, which is simultaneously measured by the area detector (PSD) and defined by Eq. (4).

In Fig. 4(a), we display an RSM recorded at the PXS in proximity to the NbO_2_ (200) and TiO_2_ (220) Bragg reflections. The intensity distribution at $\vec{G}$ of the substrate TiO_2_ illustrates the shape of the PXS's resolution area due to the high crystalline quality of the substrate. In principle, it can be approximated by two 2D-Gaussian functions that are elongated along *q_z_* and rotated by the diffraction angle $\theta_{B}$.^19^ The scanning resolution of the RSM and the small diffraction angle, however, limit the clear separation of the K_*α*_ doublet. We fit the resolution area with a single 1D Gaussian function with $\sigma_{RA,x}=6\times 10^{-4}$ Å^–1^ and $\sigma_{RA,z}=6\times 10^{-3}$ Å^–1^, which is rotated by $\theta_{B}-3{}^{\circ}$ to account for splitting.

The intensity distribution in proximity to the NbO_2_ reflection consists of two components that are indicated by the black contour lines corresponding to different intensities in Fig. 4(a). The two components have been discussed in detail in Sec. III B, and here we see the instrumental broadening compared to synchrotron setups. Clearly, the narrow component is rotated, compared to the measurement at the synchrotron, see Fig. 2, exhibiting a similar shape as the substrate reflection, i.e., it is also limited by the resolution area of the setup.

Upon femtosecond laser excitation, the measured RSM changes, as phonons are coherently and incoherently excited, triggering a long-lasting thermal expansion and picosecond strain pulses.^29^ Since the thermal expansion coefficient of NbO_2_ perpendicular to the surface is positive above 300 K, the generated strain is positive, which leads to a shift of $\vec{G}$ to smaller *q_z_*.^27^ The red contour lines in Fig. 4 indicate the shift $\Delta q_{z}$ after a pump–probe delay of 7 ps compared to the RSM before excitation (black contour lines). The projections onto the *q_x_* and *q_z_* axes confirm a shift exclusively along *q_z_*. Also, the contour lines of the substrate reflection do not change since 7 ps is not enough time to transfer significant amounts of energy into the substrate by heat diffusion. Energy deposition inside the substrate by the initial excitation pulse is extremely unlikely as the bandgap of TiO_2_ with over 3 eV exceeds the used pump photon energy of 1.55 eV.^30^ The dynamics of both components from the twofold reflection of NbO_2_ are the same since the pump and probe spots of the UXRD measurement with diameters of 1 and 0.3 mm, respectively, average over many of the small, equally strained domains with large and small coherence lengths, as described in the case of static heating.

**FIG. 5.**
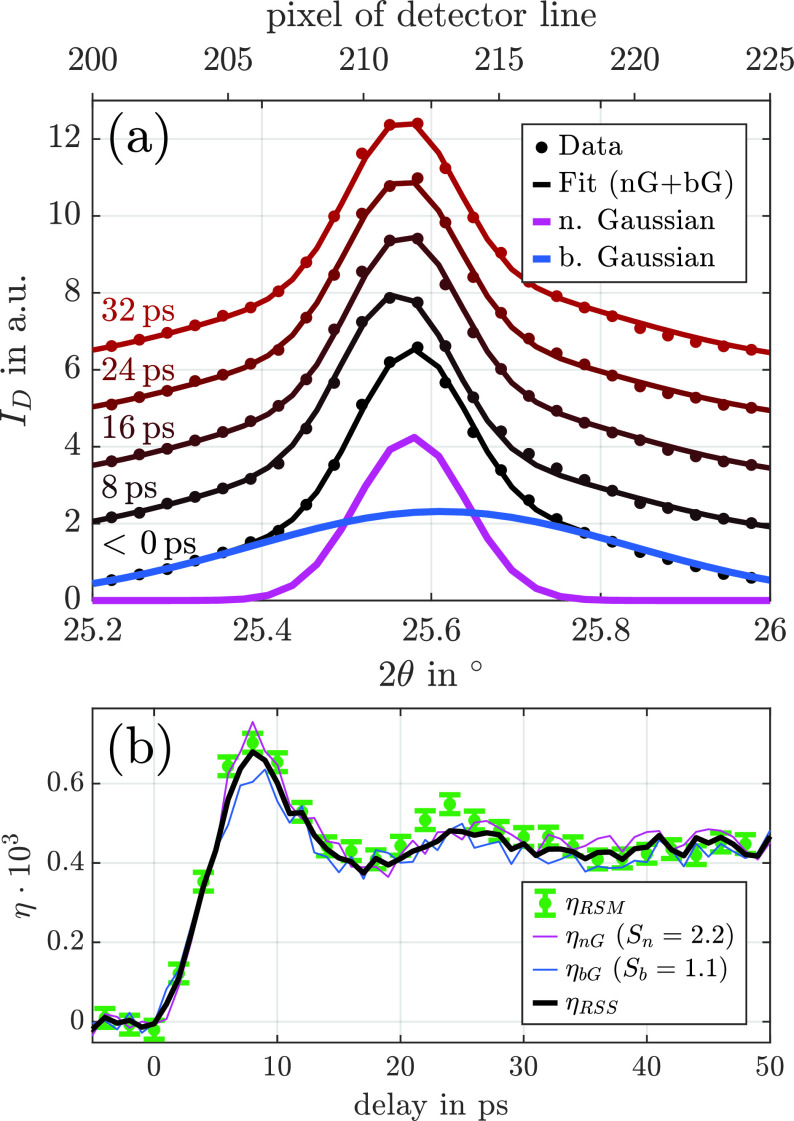
(a) Intensity distribution on the detector line before excitation (black) and after several ps delays (black to red). The data are fitted with the sum of two Gaussian functions, a narrow Gaussian (nG, magenta) and a broad Gaussian (bG, blue). (b) Average strain inside the NbO_2_ layer upon femtosecond laser excitation. The strain is determined with single Gaussian fits of the full reciprocal space map projection onto the *q_z_* axis (green dots). A sum of two Gaussian functions is used to fit the reciprocal space slicing data (black). The latter is the average of the strain from the narrow Gaussian fit (magenta) and the broad Gaussian fit (blue), calculated via Eq. (3) with, respectively, different scaling factors $S_{n}=2.2$ and $S_{b}=1.1$.

To quantitatively determine the strain response of the thin NbO_2_ layer after femtosecond laser excitation, we first recorded full reciprocal space maps and later slices of the reciprocal space as a function of the pump–probe delay under identical measurement conditions. The intensity distributions of the reciprocal space maps were projected onto the *q_z_* axis, as shown in Fig. 4(b) to extract the shift $\Delta q_{z}$ for each delay of pump and probe pulses. This is done with a single Gaussian fit since the twofold nature of the NbO_2_ reflection manifests itself only in the *q_x_* direction, not in *q_z_* (see Fig. 4). Via the scaled derivative of Bragg's law, i.e., the right half of Eq. (3), the strain was calculated and is plotted in Fig. 5(b) as green circles (*η_RSM_*). The error bars indicate the uncertainty of the strain assessment given by the standard deviation of the strain for negative delays.

In contrast to the full reciprocal space mapping approach, the diffraction geometry is not changed during the reciprocal space slicing. The detector measures the intensity of the reciprocal space slice along the 2θ-axis indicated by the white line in Fig. 4(a). The intensity distribution before excitation is displayed in Fig. 5(a). The sum of two Gaussian functions (black) fits the data.

According to Eq. (3), we calculate the strain η(t) from the shifts of the two contributions to the intensity distribution on the detector Δ2θ(t) separately, where *S* is set to $S_{n}=2.2$ and $S_{b}=1.1$. The resulting strain transients *η_nG_* and *η_bG_* are displayed in Fig. 5(b) as magenta and blue lines, respectively. The scaling factors are calculated with Eq. (8) using the widths in the reciprocal space measured at the synchrotron and the widths of the resolution area of the PXS setup. To account for the Cu $K_{\alpha }$ doublet, we applied an angular offset of 3° to the diffraction angle. The transformation of the measured angles into reciprocal space is sensitive to experimental parameters such as the sample-detector distance, the relation between the 2θ and pixel axis, and the alignment of the sample to the rotation center of the goniometer.

We observe that the broad Gaussian part (blue) shifts significantly more on the detector after excitation than the narrow Gaussian part (magenta), i.e., ${\left(\Delta 2\theta \right)}_{b}>{\left(\Delta 2\theta \right)}_{n}$. Only the correct scaling of the shifts reveals a qualitative and quantitative agreement of the strain value deduced from the change in the diffraction pattern [thin blue and magenta lines in Fig. 5(b)]. The strain dynamics observed using the reciprocal space slicing technique also agree with the strain determined via conventional full reciprocal space mapping.

The presented strain response in NbO_2_ upon femtosecond laser excitation corresponds well to the standard model of laser-excited thin film transducers probed by x-ray diffraction.^31^ A bipolar strain wave is launched, which traverses and leaves the NbO_2_ thin film within the first 20 ps. The average layer strain rises while the layer is expanding, and the compressive leading half of the bipolar wave is ejected into the substrate. When the trailing expansive half of the coherent phonon wavepacket exits the layer toward the substrate, the strain level decreases to 0.04%, which is 2/3 of the maximum strain as it is expected for a perfectly impedance matched layer. In this case, we observe an additional local maximum at 25 ps, which corresponds to the expansive strain of the partly reflected strain wave from the layer interface. The residual expansion beyond 35 ps originates solely from thermal expansion and decays on a nanosecond timescale via heat diffusion. The observed timings of the strain pulse in Fig. 5(b) are consistent with the thickness and the longitudinal sound velocity of the NbO_2_ layer.

The presented example demonstrates that reciprocal space slicing yields the same quantitative and qualitative strain dynamics as conventional reciprocal space mapping does. The full reciprocal space mapping measurement took three times longer than the slicing while having only half of the delay steps scanned. Thus, the reciprocal space slicing can assess strain dynamics in thin films almost an order of magnitude faster. The slicing approach readily shows that the strain dynamics of both components of the (200) Bragg reflection in NbO_2_ have the same amplitude. In general, different samples may exhibit nano-domains that exhibit disparate strain dynamics, and the slicing technique would be able to measure this difference as long as the dynamics are one-dimensional. This illustrates an advantage of the slicing performed at the PXS compared to the synchrotron evaluation presented in Sec. III. There, it is only practically possible to determine the strain from the broad component of the Bragg reflection since the resolution area was too small and not tilted in the reciprocal space. Paradoxically, the instrumental broadening by a convergence and energy spread is beneficial for the slicing scheme for samples of high crystalline quality.

It may be important to reanalyze experimental work in the field of ultrafast x-ray diffraction and strain assessment that uses some form of reduced reciprocal space analysis.^8–10,32–48^ From the very early days of UXRD using plasma sources, the large convergence of the x-rays was used to speed up the measurements by area detectors. The correct scaling was often considered unimportant, maybe because experimental determination of the fluence introduces considerable uncertainties. In other cases, only scaled quantities were relevant. We would like to note, however, that some publications in this context use the phrase “rocking curve” for experimental conditions, where the sample is not “rocked,” but instead the convergence of the source and the area detector are used to measure different angles simultaneously. We now think that it would be good to point out the precise experimental conditions in future publications. Several publications of our group were based on UXRD measurements applying the reciprocal space slicing approach.^8–10,42–48^ We reviewed all of them and found that the claims and findings are still correct. In most cases, this is because the scaling factor is negligible due to large mosaicities of thin films and small diffraction angles. In some other cases, only the qualitative strain response is evaluated, rendering the scaling irrelevant.

## V. CONCLUSIONS

Our analysis shows that the interpretation of ultrafast x-ray diffraction experiments using RSS instead of full RSM requires quantitative characterization of the natural Bragg peak widths in reciprocal space around the investigated reciprocal lattice point. We provide formulas to calculate the scaling factor *S* that is required to quantify the strain from the shift of a Bragg peak in a reciprocal space slice, which is recorded in experiments using position-sensitive detectors. The scaling factor depends on the width of the measured intensity distributions along *q_x_* and *q_z_* in reciprocal space. This is given by the instrumental resolution area, the structural properties of the crystal investigated, and the diffraction angle. Reciprocal space slicing is an excellent method for speeding up time-resolved x-ray diffraction experiments.

The formulas for the appropriate scaling factor indicate that the slicing is generally ineffective, when *S* is very large, due to a small signal-to-noise ratio. This is the case for large diffraction angles or reflections, which are much broader along *q_z_* compared to *q_x_* because the measurable shift on the detector Δ2θ then becomes very small. Our examples show that a broad resolution area may be advantageous for rapid slicing of the reciprocal space. In a typical synchrotron experiment with negligible instrumental broadening, reflections from crystals that have a much larger coherence length along *q_x_* than along *q_z_* exhibit scaling factor *S* that can be larger than 10, so that shifts along *q_z_* yield only very small observable changes in a reciprocal space slicing experiment. For substrate-like reflections, which have large coherence lengths along both *q_x_* and *q_z_*, even tiny strains shift $\vec{G}$ along *q_z_* such that the detector only intersects the wings of the associated Bragg reflection, with considerable intensity loss. Using a convergent x-ray beam with a consequently larger resolution area prevents this at the expense of angular resolution.

We conclude that reciprocal space slicing is a useful tool for strain assessment, from the static heating to femtosecond laser excitation. It works particularly well for small scaling factors *S*, i.e., small diffraction angles and small coherence lengths in-plane, for example, thin metal films with large mosaicities. The average strain of thin layers is correctly assessed even for inhomogeneous strain patterns, although details of the strain distribution are better characterized by full reciprocal space mapping, especially in the context of phase transitions. Even transient changes of the coherence lengths, due to strongly inhomogeneous strain patterns, and the resulting changes of the Bragg reflection widths can, in principle, be incorporated by a transient scaling factor and, thus, lead to a correct strain assessment. If strong structural changes in two or three dimensions, which change the coherence volume, are expected, full reciprocal space mapping is a better alternative. We hope that our analysis will help in designing and interpreting future UXRD experiments.

## Data Availability

The data that support the findings of this study are available from the corresponding authors upon reasonable request.

## APPENDIX: CALCULATION OF THE SCALING FACTOR FOR THE PXS SETUP

With an ellipsoidal parameterization of the exponent of the Gaussian functions, the resolution area can be described by

$$\begin{array}{c} RA(q_{x},q_{z})=RA_{1}(q_{x},q_{z})+RA_{2}(q_{x},q_{z})\\ =A_{\text{RA},1}\;\text{exp}\;\left(-aq_{x}^{2}-2bq_{x}q_{z}-cq_{z}^{2}\right)\\ +A_{\text{RA},2}\;\text{exp}\;\left(-aq_{x}^{2}-2bq_{x}\left(1+\frac{\Delta k}{k_{\text{in}}}\right)q_{z}-c{\left(1+\frac{\Delta k}{k_{\text{in}}}\right)}^{2}q_{z}^{2}\right), \end{array}$$ (A1)

where $k_{\text{in}}$ is the wave vector of ${\text{K}}_{\alpha 1},\;\Delta k$ accounts for the separation, and $A_{\text{RA},1}$ and $A_{\text{RA},2}$ for the relative intensities of the K_*α*_ doublet. *a*, *b*, and *c* are parameters of the ellipsoid and are defined by

$$\begin{array}{c} a:=\frac{\;\text{cos}\;{\left(\omega \right)}^{2}}{2\sigma_{\text{RA},x}^{2}}+\frac{\;\text{sin}\;{\left(\omega \right)}^{2}}{2\sigma_{\text{RA},z}^{2}},\\ b:=-\frac{\;\text{sin}\;(\omega )}{4\sigma_{\text{RA},x}^{2}}+\frac{\;\text{sin}\;(\omega )}{4\sigma_{\text{RA},z}^{2}},\\ c:=\frac{\;\text{sin}\;{\left(\omega \right)}^{2}}{2\sigma_{\text{RA},x}^{2}}+\frac{\;\text{cos}\;{\left(\omega \right)}^{2}}{2\sigma_{\text{RA},z}^{2}}, \end{array}$$ (A2)

where $\sigma_{\text{RA},x}$ and $\sigma_{\text{RA},x}$ are the widths in *q_x_* and *q_z_* directions of the 2D Gaussian function before rotating by the angle *ω*.

The convolution of this resolution area with the reciprocal space of the specimen around $\vec{G}$ then equals the RSM measured at the PXS setup $I(q_{x},q_{z})$, as described by Eqs. (1) and (2) in Sec. II. Since convolutions are linear operations of functions, it is sufficient to evaluate the convolution integral of *RS* and the first addend *RA*_1_. The convolution of the second part of *RA* with *RS* is identical after a coordinate transformation to account for the separation by $\Delta k/k_{\text{in}}$. We find that the convolution $I_{1}(q_{x},q_{z}):=(RS*RA_{1})(q_{x},q_{z})$ is again a 2D Gaussian function. In order to derive the intensity distribution $I_{D_{1}}$ of a shifted $\vec{G}$ measured along the slice of the detector, we again substitute *q_x_* in $I_{1}(q_{x},q_{z})$ with its equivalent expression given in Eq. (4). The function is also Gaussian and can, therefore, be written as

$$\begin{array}{c} I_{D_{1}}=I_{1}(-(q_{z}-q_{z,0})\;\text{tan}\;(\theta_{B}),q_{z})\\ =A_{I_{D1}}\;\text{exp}\;\left(-\frac{{\left(q_{z}-\frac{\Delta q_{z}}{S}\right)}^{2}}{2\sigma_{I_{D1}}^{2}}\right), \end{array}$$ (A3)

where $A_{I_{D1}}$ is the amplitude and $\sigma_{I_{D1}}$ is the width of the intensity distribution of the detector projected onto the *q_z_* axis. The scaling factor *S* connects the measured *q_z_* shift on the detector ($\Delta q_{z,D}$) with the real shift $\Delta q_{z}$ of $\vec{G}$, similar to Eq. (6). In this case, *S* depends on the parameters of the resolution area as well.

## References

[1] A. Rousse , C. Rischel , and J.-C. Gauthier , “ Femtosecond x-ray crystallography,” Rev. Mod. Phys. 73, 17–31 (2001). 10.1103/RevModPhys.73.17

[2] M. Bargheer , N. Zhavoronkov , M. Woerner , and T. Elsaesser , “ Recent progress in ultrafast x-ray diffraction,” Chemphyschem 7, 783–792 (2006). 10.1002/cphc.200500591 16596604

[3] M. Chergui and A. H. Zewail , “ Electron and x-ray methods of ultrafast structural dynamics: Advances and applications,” Chemphyschem 10, 28–43 (2009). 10.1002/cphc.200800667 19130540

[4] T. Elsaesser and M. Woerner , “ Perspective: Structural dynamics in condensed matter mapped by femtosecond x-ray diffraction,” J. Chem. Phys. 140, 020901 (2014). 10.1063/1.4855115 24437858

[5] M. Kozina , M. Fechner , P. Marsik , T. van Driel , J. M. Glownia , C. Bernhard , M. Radovic , D. Zhu , S. Bonetti , U. Staub , and M. C. Hoffmann , “ Terahertz-driven phonon upconversion in SrTiO_3_,” Nat. Phys. 15, 387–392 (2019). 10.1038/s41567-018-0408-1

[6] S. Pandey , R. Bean , T. Sato , I. Poudyal , J. Bielecki , J. C. Villarreal , O. Yefanov , V. Mariani , T. A. White , C. Kupitz , M. Hunter , M. H. Abdellatif , S. Bajt , V. Bondar , A. Echelmeier , D. Doppler , M. Emons , M. Frank , R. Fromme , Y. Gevorkov , G. Giovanetti , M. Jiang , D. Kim , Y. Kim , H. Kirkwood , A. Klimovskaia , J. Knoska , F. H. M. Koua , R. Letrun , S. Lisova , L. Maia , V. Mazalova , D. Meza , T. Michelat , A. Ourmazd , G. Palmer , M. Ramilli , R. Schubert , P. Schwander , A. Silenzi , J. Sztuk-Dambietz , A. Tolstikova , H. N. Chapman , A. Ros , A. Barty , P. Fromme , A. P. Mancuso , and M. Schmidt , “ Time-resolved serial femtosecond crystallography at the european XFEL,” Nat. Methods 17, 73–76 (2020). 10.1038/s41592-019-0628-z 31740816 PMC9113060

[7] G. J. Williams , S. Lee , D. A. Walko , M. A. Watson , W. Jo , D. R. Lee , and E. C. Landahl , “ Direct measurements of multi-photon induced nonlinear lattice dynamics in semiconductors via time-resolved x-ray scattering,” Sci. Rep. 6, 39506 (2016). 10.1038/srep39506 28004757 PMC5177891

[8] A. von Reppert , L. Willig , J.-E. Pudell , S. P. Zeuschner , G. Sellge , F. Ganss , O. Hellwig , J. A. Arregi , V. Uhl\v r , A. Crut , and M. Bargheer , “ Spin stress contribution to the lattice dynamics of FePt,” Sci. Adv. 6, eaba1142 (2020). 10.1126/sciadv.aba1142 32685678 PMC7343378

[9] S. P. Zeuschner , J.-E. Pudell , A. von Reppert , M. Deb , E. Popova , N. Keller , M. Rössle , M. Herzog , and M. Bargheer , “ Measurement of transient strain induced by two-photon excitation,” Phys. Rev. Res. 2, 022013 (2020). 10.1103/PhysRevResearch.2.022013

[10] J. Pudell , A. A. Maznev , M. Herzog , M. Kronseder , C. H. Back , G. Malinowski , A. von Reppert , and M. Bargheer , “ Layer specific observation of slow thermal equilibration in ultrathin metallic nanostructures by femtosecond x-ray diffraction,” Nat. Commun. 9, 3335 (2018). 10.1038/s41467-018-05693-5 30127415 PMC6102217

[11] U. Pietsch , V. Holý , and T. Baumbach , *High-Resolution X-Ray Scattering: From Thin Films to Lateral Nanostructures*, Advanced Texts in Physics, 2nd ed. ( Springer, New York, 2004).

[12] P. F. Fewster , “ Reciprocal space mapping,” Crit. Rev. Solid State Mater. Sci. 22, 69–110 (1997). 10.1080/10408439708241259

[13] A. Kinne , M. Thoms , H. R. Ress , T. Gerhard , M. Ehinger , W. Faschinger , and G. Landwehr , “ Image plates as one-dimensional detectors in high-resolution x-ray diffraction,” J. Appl. Crystallogr. 31, 446–452 (1998). 10.1107/S002188989701947X

[14] O. Masson , A. Boulle , R. Guinebretière , A. Lecomte , and A. Dauger , “ On the use of one-dimensional position sensitive detector for x-ray diffraction reciprocal space mapping: Data quality and limitations,” Rev. Sci. Instrum. 76, 063912 (2005). 10.1063/1.1938850

[15] M. Kozina , T. Hu , J. S. Wittenberg , E. Szilagyi , M. Trigo , T. A. Miller , C. Uher , A. Damodaran , L. Martin , A. Mehta , J. Corbett , J. Safranek , D. A. Reis , and A. M. Lindenberg , “ Measurement of transient atomic displacements in thin films with picosecond and femtometer resolution,” Struct. Dyn. 1, 034301 (2014). 10.1063/1.4875347 26798776 PMC4711600

[16] M. Reinhardt and W. Leitenberger , “ Xpp: X-ray pump probe station at BESSY II,” J. Large-Scale Res. Facil. 2, 1–4 (2016). 10.17815/jlsrf-2-82

[17] A. Koc , M. Reinhardt , A. von Reppert , M. Roessle , W. Leitenberger , K. Dumesnil , P. Gaal , F. Zamponi , and M. Bargheer , “ Ultrafast x-ray diffraction thermometry measures the influence of spin excitations on the heat transport through nanolayers,” Phys. Rev. B 96, 014306 (2017). 10.1103/PhysRevB.96.014306

[18] F. Zamponi , Z. Ansari , C. Korff Schmising , P. Rothhardt , N. Zhavoronkov , M. Woerner , T. Elsaesser , M. Bargheer , T. Trobitzsch-Ryll , and M. Haschke , “ Femtosecond hard x-ray plasma sources with a kilohertz repetition rate,” Appl. Phys. A 96, 51–58 (2009). 10.1007/s00339-009-5171-9

[19] D. Schick , R. Shayduk , A. Bojahr , M. Herzog , C. von Korff Schmising , P. Gaal , and M. Bargheer , “ Ultrafast reciprocal-space mapping with a convergent beam,” J. Appl. Crystallogr. 46, 1372–1377 (2013). 10.1107/S0021889813020013

[20] J. Stoever , J. E. Boschker , S. Bin Anooz , M. Schmidbauer , P. Petrik , J. Schwarzkopf , M. Albrecht , and K. Irmscher , “ Approaching the high intrinsic electrical resistivity of nbo 2 in epitaxially grown films,” Appl. Phys. Lett. 116, 182103 (2020). 10.1063/5.0005523

[21] T. Sakata , K. Sakata , and I. Nishida , “ Study of phase transition in NbO_2_,” Phys. Status Solidi B 20, K155–K157 (1967). 10.1002/pssb.19670200263

[22] S. H. Shin , T. Halpern , and P. M. Raccah , “ High–speed high–current field switching of NbO_2_,” J. Appl. Phys. 48, 3150–3153 (1977). 10.1063/1.324047

[23] S. Kim , J. Park , J. Woo , C. Cho , W. Lee , J. Shin , G. Choi , S. Park , D. Lee , B. H. Lee , and H. Hwang , “ Threshold-switching characteristics of a nanothin-NbO_2_-layer-based Pt/NbO_2_/Pt stack for use in cross-point-type resistive memories,” Microelectron. Eng. 107, 33–36 (2013). 10.1016/j.mee.2013.02.084

[24] S. Slesazeck , H. Mähne , H. Wylezich , A. Wachowiak , J. Radhakrishnan , A. Ascoli , R. Tetzlaff , and T. Mikolajick , “ Physical model of threshold switching in NbO_2_ based memristors,” RSC Adv. 5, 102318–102322 (2015). 10.1039/C5RA19300A

[25] M. R. Beebe , J. M. Klopf , Y. Wang , S. Kittiwatanakul , J. Lu , S. A. Wolf , and R. A. Lukaszew , “ Time-resolved light-induced insulator-metal transition in niobium dioxide and vanadium dioxide thin films,” Opt. Mater. Express 7, 213 (2017). 10.1364/OME.7.000213

[26] R. K. Kirby , “ Thermal expansion of rutile from 100 to 700 °K,” J. Res. Nat. Bur. Stand. Sect. A, Phys. Chem. 71A, 363–369 (1967). 10.6028/jres.071A.041 PMC6624720 31824060

[27] J. Kaiser , D. S. Rimai , and R. J. Sladek , “ Thermal expansivity in semiconducting NbO_2_,” Solid State Commun. 32, 925–927 (1979). 10.1016/0038-1098(79)90799-3

[28] M. Bargheer , N. Zhavoronkov , R. Bruch , H. Legall , H. Stiel , M. Woerner , and T. Elsaesser , “ Comparison of focusing optics for femtosecond x-ray diffraction,” Appl. Phys. B 80, 715–719 (2005). 10.1007/s00340-005-1792-7

[29] P. Ruello and V. E. Gusev , “ Physical mechanisms of coherent acoustic phonons generation by ultrafast laser action,” Ultrasonics 56, 21–35 (2015). 10.1016/j.ultras.2014.06.004 25038958

[30] T. Guang-Lei , H. Hong-Bo , and S. Jian-Da , “ Effect of microstructure of tio 2 thin films on optical band gap energy,” Chin. Phys. Lett. 22, 1787–1789 (2005). 10.1088/0256-307X/22/7/062

[31] D. Schick , M. Herzog , A. Bojahr , W. Leitenberger , A. Hertwig , R. Shayduk , and M. Bargheer , “ Ultrafast lattice response of photoexcited thin films studied by x-ray diffraction,” Struct. Dyn. 1, 064501 (2014). 10.1063/1.4901228 26798784 PMC4714650

[32] C. Rose-Petruck , R. Jimenez , T. Guo , A. Cavalleri , C. W. Siders , F. Rksi , J. A. Squier , B. C. Walker , K. R. Wilson , and C. P. J. Barty , “ Picosecond–milliångström lattice dynamics measured by ultrafast x-ray diffraction,” Nature 398, 310–312 (1999). 10.1038/18631

[33] K. Sokolowski-Tinten , C. Blome , C. Dietrich , A. Tarasevitch , M. Horn von Hoegen , D. von der Linde , A. Cavalleri , J. Squier , and M. Kammler , “ Femtosecond x-ray measurement of ultrafast melting and large acoustic transients,” Phys. Rev. Lett. 87, 225701 (2001). 10.1103/PhysRevLett.87.225701 11736408

[34] C. v. Korff Schmising , M. Bargheer , M. Kiel , N. Zhavoronkov , M. Woerner , T. Elsaesser , I. Vrejoiu , D. Hesse , and M. Alexe , “ Strain propagation in nanolayered perovskites probed by ultrafast x-ray diffraction,” Phys. Rev. B 73, 212202 (2006). 10.1103/PhysRevB.73.212202

[35] A. Morak , T. Kämpfer , I. Uschmann , A. Lübcke , E. Förster , and R. Sauerbrey , “ Acoustic phonons in InSb probed by time-resolved x-ray diffraction,” Phys. Status Solidi B 243, 2728–2744 (2006). 10.1002/pssb.200542387

[36] H. J. Lee , J. Workman , J. S. Wark , R. D. Averitt , A. J. Taylor , J. Roberts , Q. McCulloch , D. E. Hof , N. Hur , S.-W. Cheong , and D. J. Funk , “ Optically induced lattice dynamics probed with ultrafast x-ray diffraction,” Phys. Rev. B: Condens. Matter Mater. Phys. 77, 132301 (2008). 10.1103/PhysRevB.77.132301

[37] M. Nicoul , U. Shymanovich , A. Tarasevitch , D. von der Linde , and K. Sokolowski-Tinten , “ Picosecond acoustic response of a laser-heated gold-film studied with time-resolved x-ray diffraction,” Appl. Phys. Lett. 98, 191902 (2011). 10.1063/1.3584864

[38] F. Quirin , M. Vattilana , U. Shymanovich , A.-E. El-Kamhawy , A. Tarasevitch , J. Hohlfeld , D. von der Linde , and K. Sokolowski-Tinten , “ Structural dynamics in FeRh during a laser-induced metamagnetic phase transition,” Phys. Rev. B 85, 020103(R) (2012). 10.1103/PhysRevB.85.020103

[39] T. G. White , P. Mabey , D. O. Gericke , N. J. Hartley , H. W. Doyle , D. McGonegle , D. S. Rackstraw , A. Higginbotham , and G. Gregori , “ Electron-phonon equilibration in laser-heated gold films,” Phys. Rev. B: Condens. Matter Mater. Phys. 90, 014305 (2014). 10.1103/PhysRevB.90.014305

[40] R. Li , H. E. Elsayed-Ali , J. Chen , D. Dhankhar , A. Krishnamoorthi , and P. M. Rentzepis , “ Ultrafast time-resolved structural changes of thin-film ferromagnetic metal heated with femtosecond optical pulses,” J. Chem. Phys. 151, 124702 (2019). 10.1063/1.5111578 31575190

[41] M. Afshari , P. Krumey , D. Menn , M. Nicoul , F. Brinks , A. Tarasevitch , and K. Sokolowski-Tinten , “ Time-resolved diffraction with an optimized short pulse laser plasma x-ray source,” Struct. Dyn. 7, 014301 (2020). 10.1063/1.5126316 31934600 PMC6941949

[42] M. Sander , A. Koc , C. T. Kwamen , H. Michaels , A. v. Reppert , J. Pudell , F. Zamponi , M. Bargheer , J. Sellmann , J. Schwarzkopf , and P. Gaal , “ Characterization of an ultrafast bragg-switch for shortening hard x-ray pulses,” J. Appl. Phys. 120, 193101 (2016). 10.1063/1.4967835

[43] A. von Reppert , J. Pudell , A. Koc , M. Reinhardt , W. Leitenberger , K. Dumesnil , F. Zamponi , and M. Bargheer , “ Persistent nonequilibrium dynamics of the thermal energies in the spin and phonon systems of an antiferromagnet,” Struct. Dyn. 3, 054302 (2016). 10.1063/1.4961253 27679803 PMC5018005

[44] A. von Reppert , R. M. Sarhan , F. Stete , J. Pudell , N. Del Fatti , A. Crut , J. Koetz , F. Liebig , C. Prietzel , and M. Bargheer , “ Watching the vibration and cooling of ultrathin gold nanotriangles by ultrafast x-ray diffraction,” J. Phys. Chem. C 120, 28894–28899 (2016). 10.1021/acs.jpcc.6b11651

[45] A. von Reppert , L. Willig , J.-E. Pudell , M. Rössle , W. Leitenberger , M. Herzog , F. Ganss , O. Hellwig , and M. Bargheer , “ Ultrafast laser generated strain in granular and continuous FePt thin films,” Appl. Phys. Lett. 113, 123101 (2018). 10.1063/1.5050234

[46] S. P. Zeuschner , T. Parpiiev , T. Pezeril , A. Hillion , K. Dumesnil , A. Anane , J. Pudell , L. Willig , M. Rössle , M. Herzog , A. von Reppert , and M. Bargheer , “ Tracking picosecond strain pulses in heterostructures that exhibit giant magnetostriction,” Struct. Dyn. 6, 024302 (2019). 10.1063/1.5084140 31041360 PMC6447272

[47] A. von Reppert , M. Mattern , J.-E. Pudell , S. P. Zeuschner , K. Dumesnil , and M. Bargheer , “ Unconventional picosecond strain pulses resulting from the saturation of magnetic stress within a photoexcited rare earth layer,” Struct. Dyn. 7, 024303 (2020). 10.1063/1.5145315 32232076 PMC7101248

[48] J.-E. Pudell , M. Mattern , M. Hehn , G. Malinowski , M. Herzog , and M. Bargheer , “ Heat transport without heating?—An ultrafast x-ray perspective into a metal heterostructure,” Adv. Funct. Mater. 30, 2004555 (2020). 10.1002/adfm.202004555

